# Sensitivity to tumor development by TALEN-mediated Trp53 mutant genes in the susceptible FVB/N mice and the resistance C57BL/6 mice

**DOI:** 10.1186/s42826-021-00107-y

**Published:** 2021-11-29

**Authors:** Woo Bin Yun, Ji Eun Kim, Mi Lim Lee, Jun Young Choi, Jin Ju Park, Bo Ram Song, Byeong Cheol Kang, Ki Taek Nam, Han-Woong Lee, Dae Youn Hwang

**Affiliations:** 1 Department of Biomaterials Science (BK21 FOUR Program), College of Natural Resources and Life Science, Pusan National University, 50 Cheonghak-ri, Samnangjin-eup, Miryang-si, Gyeongsangnam-do 50463 Korea; 2 Department of Experimental Animal Research, Biomedical Research Institute, Seoul National University College of Medicine, Seoul, 03080 Korea; 3 Severance Biomedical Science Institute, College of Medicine, Yonsei University, Seoul, 03722 South Korea; 4 Department of Biochemistry, College of Life Science and Biotechnology, Yonsei University, Seoul, 03722 Korea

**Keywords:** Trp53, Tumorigenesis, TALEN, FVB/N, C57BL/6

## Abstract

**Background:**

This study was undertaken to compare the sensitivities of mice strains during tumor induction by transcription activator-like effector nucleases (TALEN)-mediated Trp53 mutant gene. Alterations of their tumorigenic phenotypes including survival rate, tumor formation and tumor spectrum, were assessed in FVB/N-Trp53^em2Hwl^/Korl and C57BL/6-Trp53^em1Hwl^/Korl knockout (KO) mice over 16 weeks.

**Results:**

Most of the physiological phenotypes factors were observed to be higher in FVB/N-Trp53^em2Hwl^/Korl KO mice than C57BL/6-Trp53^em1Hwl^/Korl KO mice, although there were significant differences in the body weight, immune organ weight, number of red blood cells, mean corpuscular volume (MCV), mean corpuscular hemoglobin (MCH), mean corpuscular hemoglobin concentration (MCHC), platelet count (PLT), total bilirubin (Bil-T) and glucose (Glu) levels in the KO mice relative to the wild type (WT) mice. Furthermore, numerous solid tumors were also observed in various regions of the surface skin of FVB/N-Trp53^em2Hwl^/Korl KO mice, but were not detected in C57BL/6-Trp53^em1Hwl^/Korl KO mice. The most frequently observed tumor in both the Trp53 KO mice was malignant lymphoma, while soft tissue teratomas and hemangiosarcomas were only detected in the FVB/N-Trp53^em2Hwl^/Korl KO mice.

**Conclusions:**

Our results indicate that the spectrum and incidence of tumors induced by the TALEN-mediated Trp53 mutant gene is greater in FVB/N-Trp53^em2Hwl^/Korl KO mice than C57BL/6-Trp53^em1Hwl^/Korl KO mice over 16 weeks.

**Supplementary Information:**

The online version contains supplementary material available at 10.1186/s42826-021-00107-y.

## Background

Generally referred to as p53, the Trp53 proteins act as tumor suppressors in multicellular organisms and are encoded by homologous genes in various organisms, such as Trp53 (in mice) and TP53 (in humans) [1, 2]. This protein prevents tumor cell growth at several points during the malignant process when it is under many types of stress, including DNA damage, oncogene activation, hypoxia, telomere attrition and deficiency of normal growth signal [3]. The activated Trp53 protein is capable of inducing various cellular responses including apoptosis, cell cycle arrest, DNA repair, differentiation, senescence and inhibition of angiogenesis [4, 5]. During inhibition of tumor growth, Trp53 plays the role of a sequence-specific transcription factor, mediating the activation or inhibition of target genes [6, 7]. Therefore, a mutation or allelic loss of the Trp53 gene induces the formation of tumors in the brain, lung, liver, breast, colon, esophagus, bladder and ovary of humans [8].

Numerous Trp53 KO mice containing large deletions of the Trp53 gene have been produced to investigate the role of Trp53 during the developmental process or tumor formation in mammals. Most of these mice showed similar phenotypes as an effective result of the null alleles. The first Trp53 KO mice were generated by the recombination of intron 4 and exon 5 in C57BL/6 mice using embryonic stem cell (ESC) targeting techniques. At 15 to 25 weeks of age, the homozygote variants of these mice were found to be dramatically susceptible to the development of multiple tumor types, and malignant lymphoma and sarcomas were frequently observed in the thymus and major visceral organs of these animals [9]. Similar tumors were also observed in KO mice having the exon 2 or exon 2–6 deletion in C57BL/6 mice. Spontaneous lymphomas predominantly developed in most KO animals with the above deletion, whereas other tumor types, including sarcomas and teratomas, occurred randomly [10–12]. Furthermore, BALB/cByJ Trp53 KO mice having deletion of exons 2–6 in the Trp53 gene exhibited lymphomas, sarcomas and mammary tumors, although lymphomas being the most frequent type of tumors [13]. Moreover, lymphomas, sarcomas and teratomas were also detected in 129S1/SvImJ and C57BL/6 × 129S1/SvImJ mice having the exon 4–5 deletion of the Trp53 gene [14, 15]. However, the comparison of tumorigenic sensitivity to TALEN-mediated Trp53 mutant gene in the tumor susceptible FVB/NJ mice and resistant C57BL/6 mice have never been considered, although several comparative studies have compared the factors affecting cancer incidence in Trp53 deficient mice [14, 15].

**Fig. 1:**
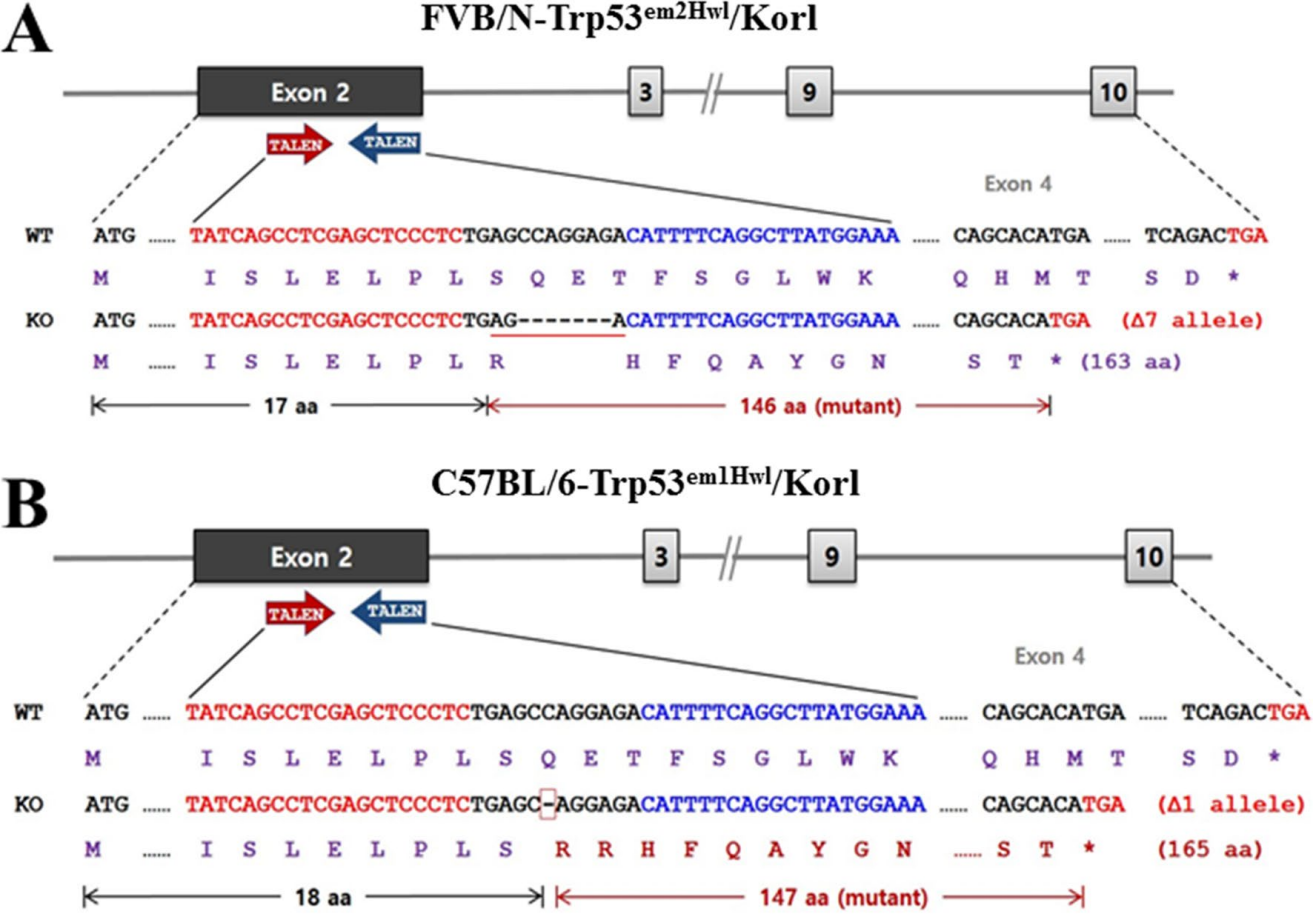
Trp53 gene deletion. After the design and synthesis of two highly active TALENs specific to exon 2 of Trp53, each TALEN mRNA was injected into the cytoplasm of mouse pronuclear-stage embryos to produce FVB/N-Trp53^em2Hwl^/Korl (**A**) and C57BL/6-Trp53^em1Hwl^/Korl (**B**) KO mice deficient in p53

This study was therefore undertaken to compared the tumorigenic phenotypes of FVB/N-Trp53^em2Hwl^/Korl and C57BL/6-Trp53^em1Hwl^/Korl KO mice encompassing the TALEN-mediated Trp53 mutant gene (Fig. 1). Our results provide additional evidence that the FVB/N mice have high susceptibility to TALEN-mediated Trp53 mutant gene during tumorigenesis, as compared to C57BL/6 mice.

## Results

### Susceptibility to body and organs weight between FVB/N-Trp53^em2Hwl^/Korl and C57BL/6-Trp53^em1Hwl^/Korl KO mice

**Fig. 2:**
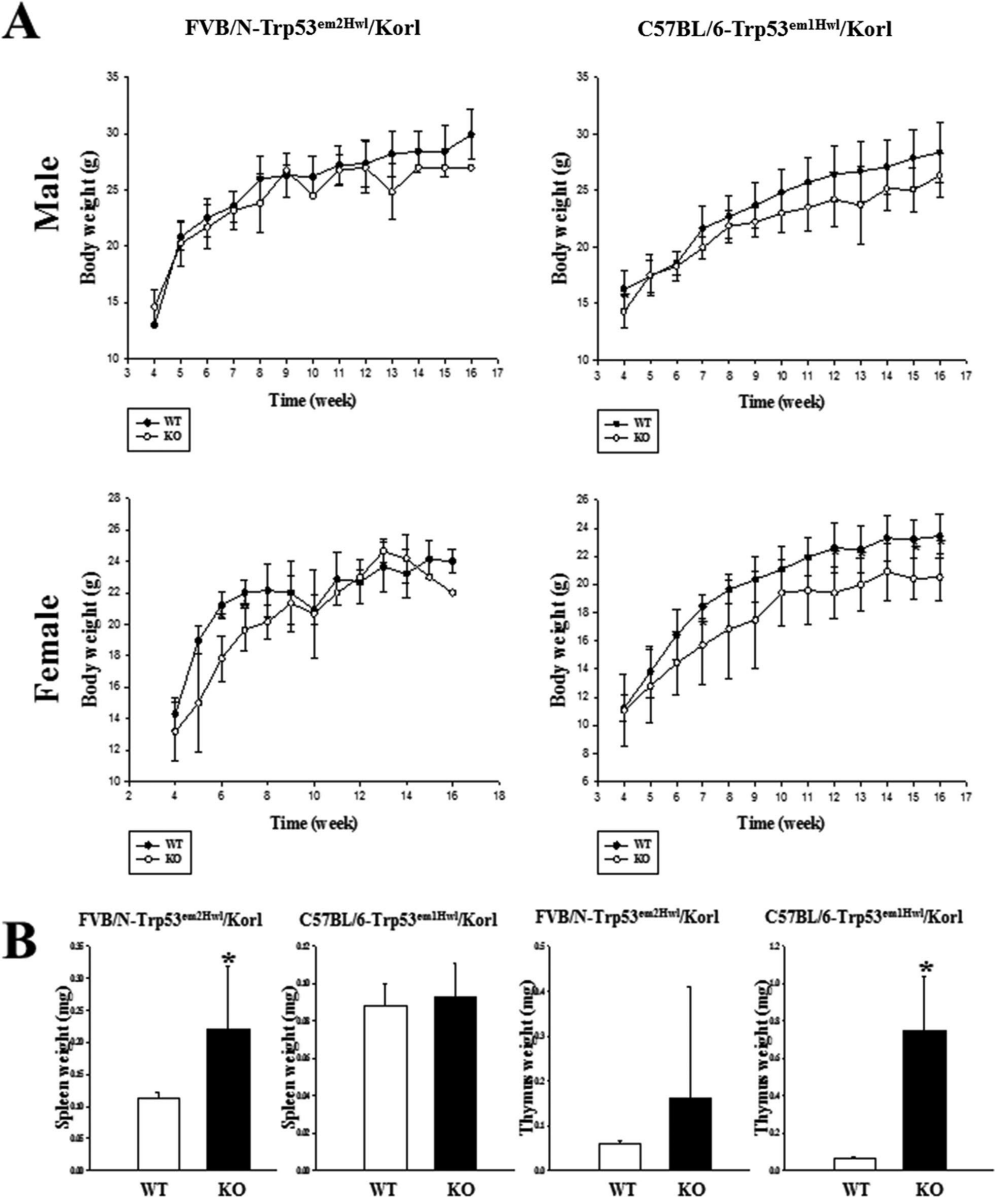
Changes in the body weight and organ weight of FVB/N-Trp53^em2Hwl^/Korl (n = 13) and C57BL/6-Trp53^em1Hwl^/Korl (n = 16) KO mice. Throughout the experimental period, the body weights (**A**) of subset groups were measured using an electrical balance, as were the weight of the spleen and thymus (**B**). The data shown represent the means ± SD of three replicates. *, *P* < 0.05 compared with the WT mice

To compare the susceptibility in the body and organs weight of the two novel models with TALEN-mediated Trp53 gene deficiency, we measured daily changes of the body weight in FVB/N-Trp53^em2Hwl^/Korl and C57BL/6-Trp53^em1Hwl^/Korl KO mice from weeks 4 to 16. We observed significant changes in the body weight of C57BL/6-Trp53^em1Hwl^/Korl KO mice. The body weight of both genders was also lower in the C57BL/6-Trp53^em1Hwl^/Korl KO mice than that observed in WT mice, although their statistical significance was only apparent in the female group (Fig. 2A). However, FVB/N-Trp53^em2Hwl^/Korl KO mice showed no significant difference in the body weight of male and female mice, except at a few time points (Fig. 2A). We also observed significant differences in organs weight. Of the nine organs considered, only the spleen weight was dramatically increased FVB/N-Trp53^em2Hwl^/Korl KO mice compared with WT mice, while the thymus weight was significantly increased in C57BL/6-Trp53^em1Hwl^/Korl KO mice (Fig. 2B). These findings suggest that the susceptibility to body weight was higher in C57BL/6-Trp53^em1Hwl^/Korl KO mice than FVB/N-Trp53^em2Hwl^/Korl KO mice.

### Differences in the blood and serum components of FVB/N-Trp53^em2Hwl^/Korl and C57BL/6-Trp53^em1Hwl^/Korl KO mice

**Fig. 3:**
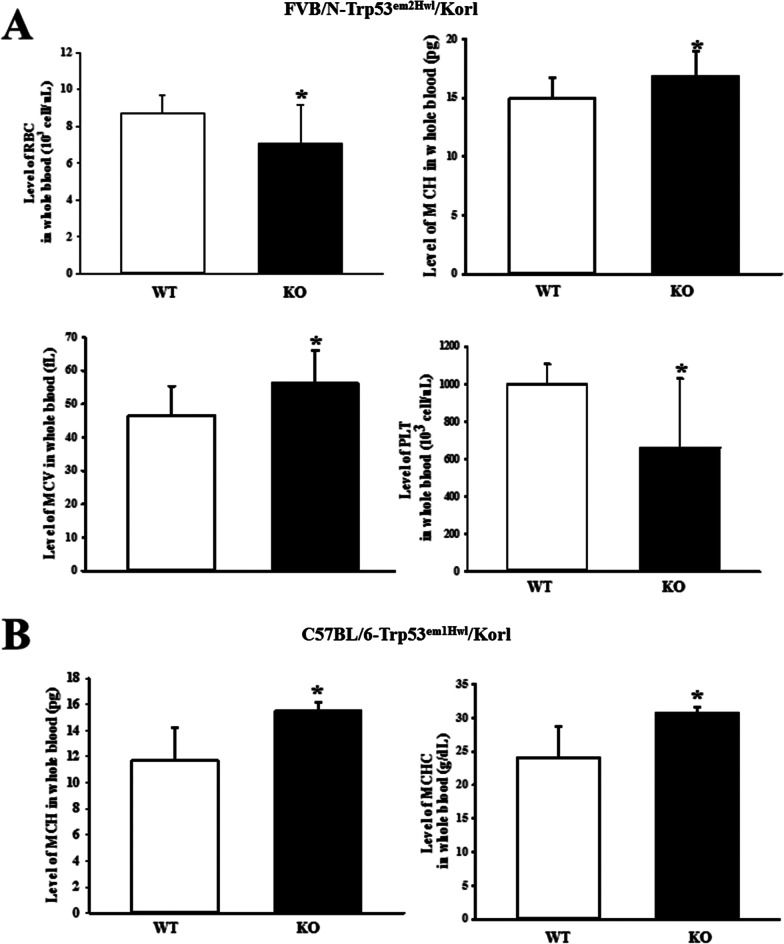
Alteration of blood parameters in FVB/N-Trp53^em2Hwl^/Korl (n = 13) (**A**) and C57BL/6-Trp53^em1Hwl^/Korl (n = 16) (**B**) KO mice. The concentrations of 12 key factors were measured in the blood of subset groups using an automatic cell counter. The data shown represent the means ± SD of three replicates. *, *P* < 0.05 compared with the WT mice

**Fig. 4:**
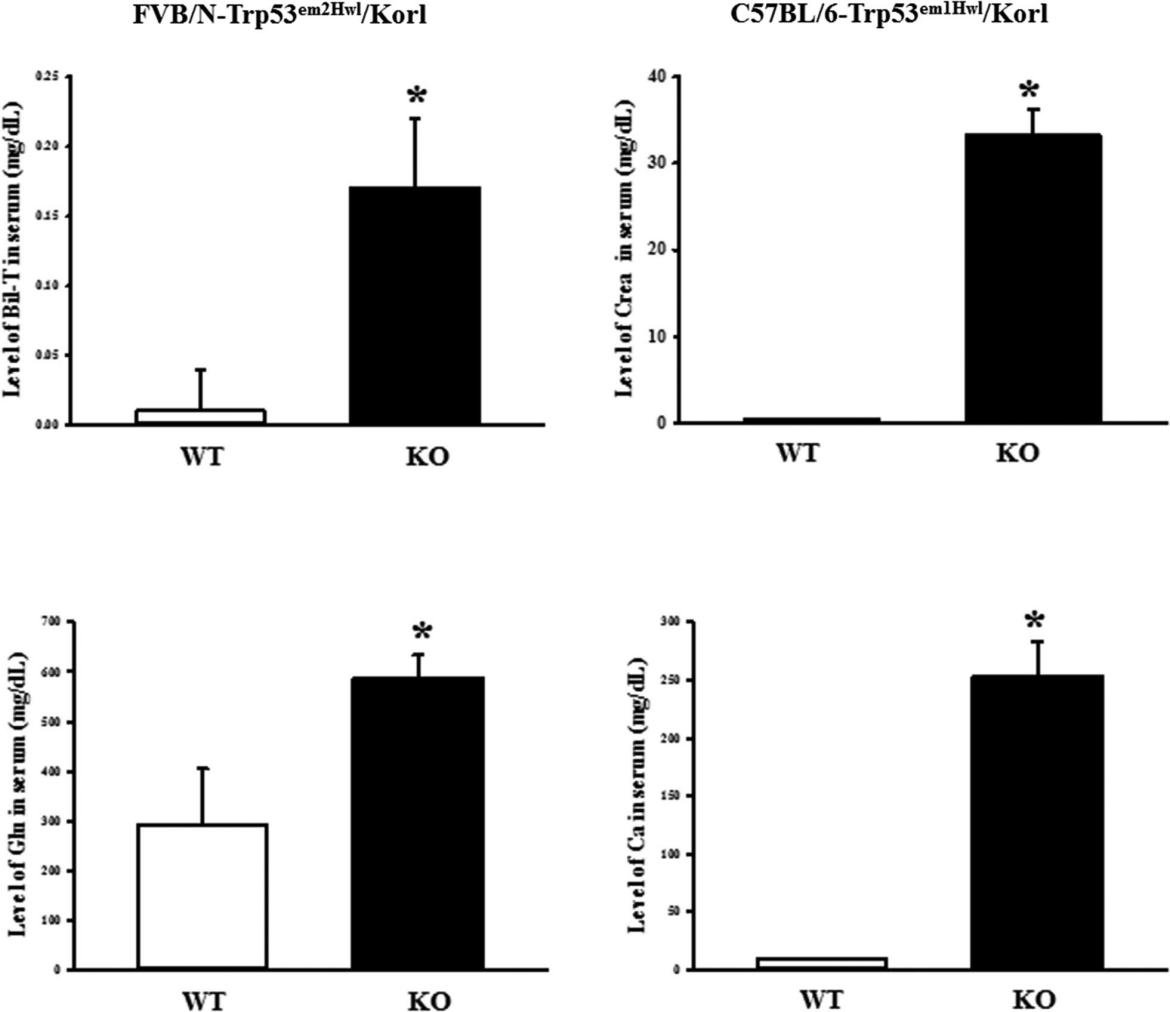
Alteration of four serum parameters in FVB/N-Trp53^em2Hwl^/Korl (n = 13) and C57BL/6-Trp53^em1Hwl^/Korl (n = 16) KO mice. The concentration of 13 key factors were measured in the serum of subset groups using an automatic serum analyzer. The data shown represent the means ± SD of three replicates. *, *P* < 0.05 compared with the WT mice

To compare the variations in the blood and serum composition of the two models with TALEN-mediated deficiency of the Trp53 gene, the level of each component in the blood and serum was measured at 16 weeks of age in FVB/N-Trp53^em2Hwl^/Korl and C57BL/6-Trp53^em1Hwl^/Korl KO mice. Blood analysis revealed enhanced levels of MCV and MHC in FVB/N-Trp53^em2Hwl^/Korl KO mice compared to the WT mice, whereas levels of RBC and platelets were decreased in the same group (Fig. 3A). However, the levels of MCH and MCHC were higher in C57BL/6-Trp53^em1Hwl^/Korl KO mice in the WT (Fig. 3B). Serum biochemical analysis revealed significant changes in 4 of the 13 factors compared between WT and KO mice. The levels of Crea and Ca were 6695% and 2610% higher in C57BL/6-Trp53^em1Hwl^/Korl KO mice than in WT mice, whereas levels of Bil-T and Glu increased by 1600% and and 101% in FVB/N-Trp53^em2Hwl^/Korl KO mice relative to WT mice (Fig. 4). These results indicate that TALEN-mediated deficiency of Trp53 genes generates significant alterations of MCV, MHC, MCHC, RBC and PLT levels in blood, as well as Crea, Bil-T, Ca and Glu levels in serum.

### Differences in the survival rate and tumorigenicity of FVB/N-Trp53^em2Hwl^/Korl and C57BL/6-Trp53^em1Hwl^/Korl KO mice

**Fig. 5:**
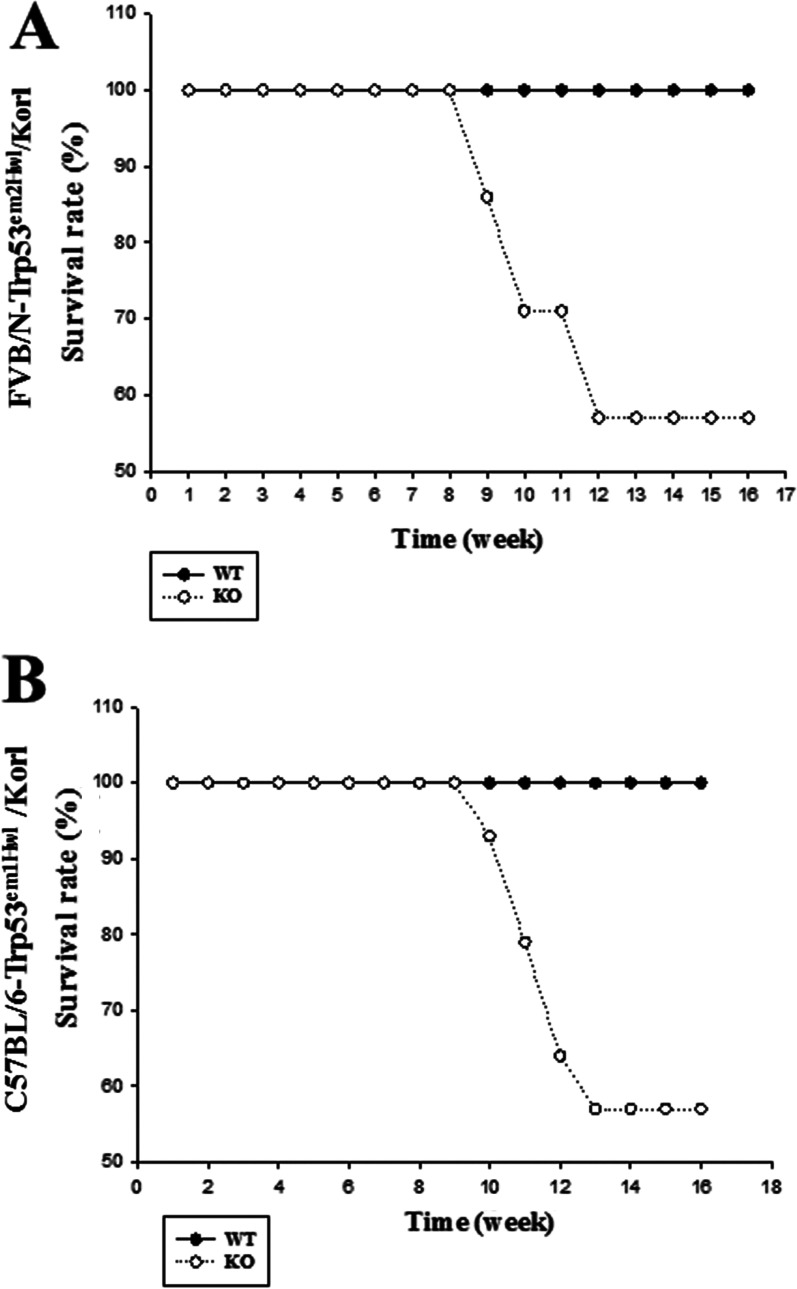
Differences in the survival rate of FVB/N-Trp53^em2Hwl^/Korl (**A**) and C57BL/6-Trp53^em1Hwl^/Korl (**B**) KO mice. Viability of 14 WT (dark circle) and 14 KO mice (white circle) monitored for 16 weeks

Finally, we compared changes in the survival rate and tumor development of the two different models containing TALEN-mediated deficiency of Trp53 gene. The survival rate of KO mice decreased rapidly at 8–10 weeks of age, whereas the WT mice maintained a consistent survival rate. Additionally, there was no significant difference in these alteration patterns was observed in the C57BL/6-Trp53^em1Hwl^/Korl and FVB/N-Trp53^em2Hwl^/Korl KO mice (Fig. 5). These results indicate that deficiency of the Trp53 protein can decrease the survival rate of both FVB/N-Trp53^em2Hwl^/Korl and C57BL/6-Trp53^em1Hwl^/Korl KO mice, regardless of the location of TALEN-mediated deletions in the Trp53 gene.

**Fig. 6:**
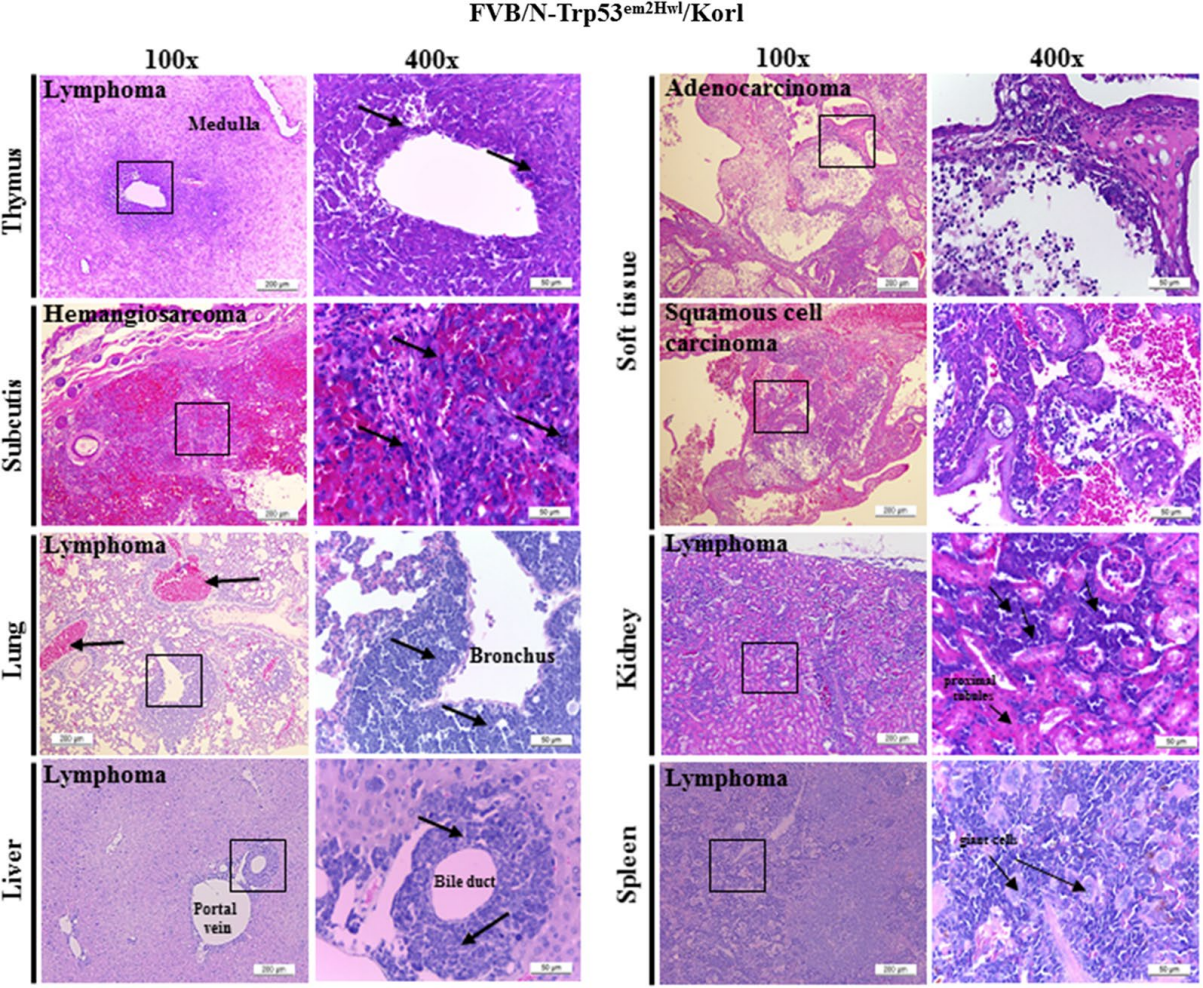
Histopathology of tumors formed in various organs of FVB/N-Trp53^em2Hwl^/Korl KO mice. H&E stained sections of each organ and solid tumor from the FVB/N-Trp53^em2Hwl^/Korl KO mice were observed at 100× (left column) and 400× (right column) using a light microscope. Rectangles in left column are magnified in the right column

**Fig. 7:**
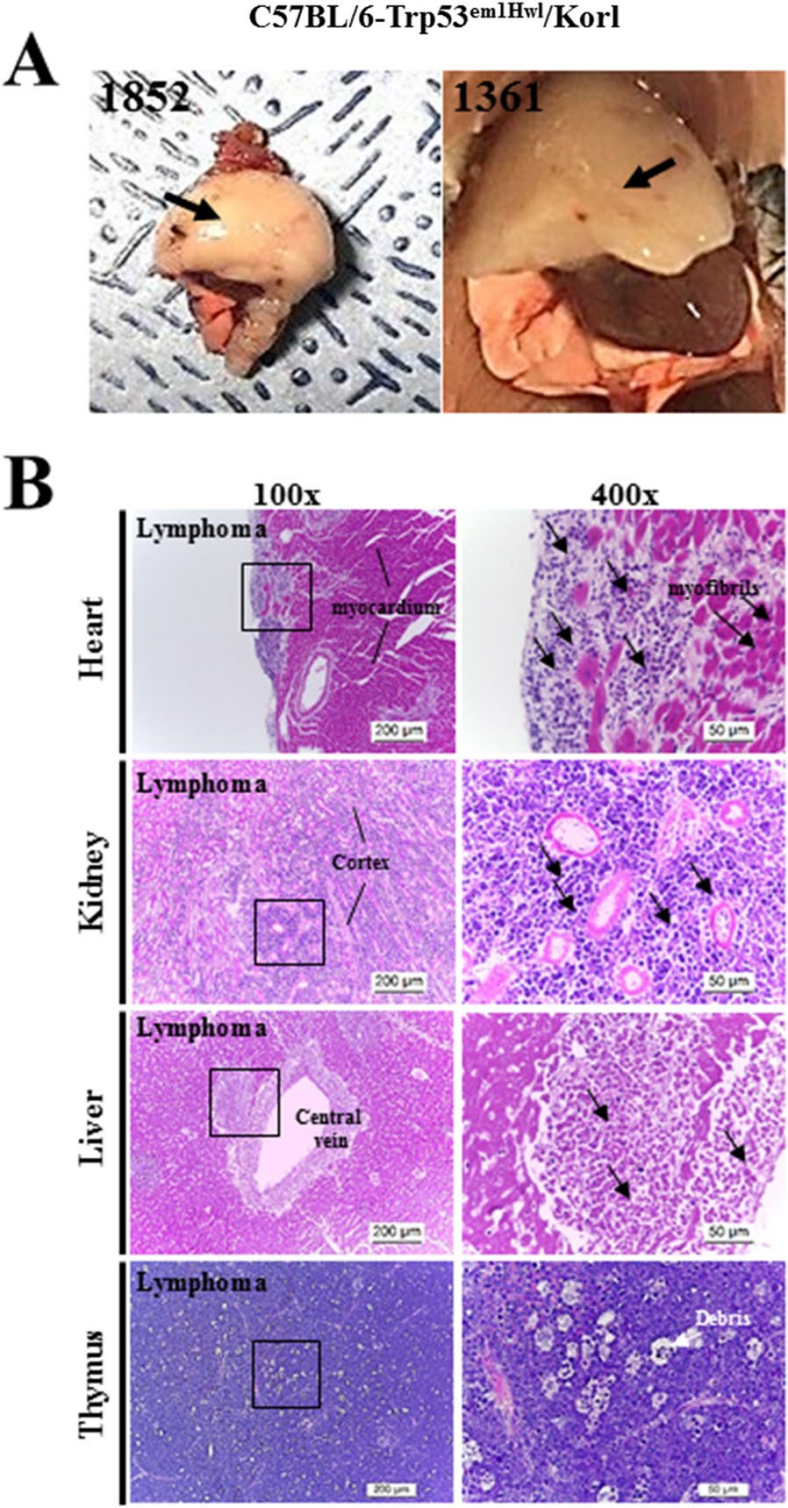
Morphology of thymus (**A**) and histopathology (**B**) of tumors formed in various organs of C57BL/6-Trp53^em1Hwl^/Korl KO mice. A large thymus was observed in the thoracic cage of two C57BL/6-Trp53^em1Hwl^/Korl KO mice. H&E stained sections of each organ and solid tumors from C57BL/6-Trp53^em1Hwl^/Korl KO mice were observed at 100× (left column) and 400× (right column) using a light microscope. Rectangles in the left column are magnified in the right column

However, a significant difference was observed in tumor development. Specifically, we observed the formation of small tumors on the body surface was observed in various regions including the eye, mouth, testis, anus, arms and back of FVB/N-Trp53^em2Hwl^/Korl KO mice, with the largest tumors being formed on the arms or backs (Fig. 6 and Table 1). No specific solid tumors were detected on the skin surface of C57BL/6-Trp53^em1Hwl^/Korl KO mice, although tumors were detected in some internal organs. Furthermore, histological analysis revealed the presence of three types of tumors, namely malignant lymphoma, teratoma and hemangiosarcoma. The most commonly occurring tumor in FVB/N-Trp53^em2Hwl^/Korl KO mice was malignant lymphoma, which was detected in the liver, lung, thymus, kidney, spleen and soft tissue; lymphocytes with condensed chromatin, irregular nuclei and less cytoplasm were concentrated around the bronchiole and blood vessels of the lung, portal triad of the liver, and blood vessels of the thymus (Fig. 6 and Table 2). In C57BL/6-Trp53^em1Hwl^/Korl KO mice, abnormal lymphocytes were primarily distributed in the myocardium of the heart, cortex of the kidney, and central vein of the liver and thymus. Moreover, common forms of teratoma with condensed chromatin and irregular nuclei were detected around the mucous membrane of soft tissues, while hemangiosarcomas were concentrated around the blood vessels of the subcutis in FVB/N-Trp53^em2Hwl^/Korl KO mice (Fig. 7 and Table 2). The results of this study suggest that the three major tumors reported in Trp53 KO mice can be successfully induced by TALEN-mediated deficiency of the Trp53 gene in FVB/N-Trp53^em2Hwl^/Korl KO mice, although only C57BL/6-Trp53^em1Hwl^/Korl KO mice showed malignant lymphoma.

**Table 1:** Tumor detection site on the skin surface of FVB/N-Trp53^em2Hwl^/Korl KO mice

Tumor detection site	Number of animals (n = 12)
Eye	1
Mouth	2
Testis	2
Anus	1
Arm or back	6

**Table 2:** Tumor incidence on C57BL/6-Trp53^emlHwl^/Korl and FVB/N-Trp53^em2Hwl^/Korl KO mice

Type of KO animal	Tumor type	Lesion tissue	Incidence
FVB/N-Trp53^em2Hwl^/Korl (n = 13)	Malignant lymphoma	Liver	2/13
		Lung	3/13
		Thymus	1/13
		Kidney	2/13
		Soft tissue	1/13
		Spleen	3/13
	Teratoma	Soft tissue	2/13
	Hemangiosarcoma	Soft tissue (subcutis)	1/13
C57BL/6-Trp53^emlHwl^/Korl (n = 16)	Malignant lymphoma	Liver	6/16
		Lung	1/16
		Kidney	4/16
		Soft tissue	1/16
		Spleen	2/16
		Brain	1/16

## Discussion

As compared to chemically induced models and xenograft models for cancer, genetically engineered mice (GEM) have the ability to accurately mimic the histology and biological behavior of human cancer [15]. Most of these models exhibit various hallmarks for human cancer including angiogenesis, stromal interaction, histopathological structure and genetic abnormalities [15]. However, despite their significant impacts, the effects of the TALEN-mediated deficiency of Trp53 gene on each model were never considered an important factor in studies using the GEM cancer model [16]. Therefore, the current study was undertaken to compare the tumorigenic phenotypes, including survival rate and tumor development, of two Trp53 KO mice containing the TALEN-mediated Trp53 mutant gene. Our results reveal that the most pronounced effects of the TALEN-mediated deficiency of Trp53 gene are observed in tumor development. Three types of tumors (malignant lymphoma, teratoma and hemangiosarcoma) were clearly observed in FVB/N-Trp53^em2Hwl^/Korl KO mice. Taken together, these results indicate that FVB/N-Trp53^em2Hwl^/Korl KO mice should be considered as appropriate candidates for therapeutic studies and identification of therapeutic drugs.

In most cases, GEMs are produced in embryonic stem cells (129S1/SvImJ and C57BL/6) or in mouse zygotes (FVB/N, C57BL/6 and SJL/J) [17]. Trp53 deficiency was generated in mice with varied genetic backgrounds, including C57BL/6, Balb/cByJ, 129S1/SvImJ, and C57BL/6 × 129S1/SvImJ mixed mice using the gene targeting method. A large number of off-spring were produced using the C57BL/6 variant, and exhibited a similar tumor type (lymphoma and sarcoma) [9–12]. However, one BALB/cByJ mouse developed lymphoma, sarcoma and mammary tumors, while 2 mice with a 129S1/SvImJv and C57BL/6 × 129/SvImJ mixed background formed lymphoma, sarcoma and teratoma [13, 14, 18]. In our study, the FVB/N background mice were first used to produce KO mice containing TALEN-mediated deficiency of the Trp53 gene. Three types of tumors (malignant lymphoma, teratoma and hemangiosarcoma) were detected in various tissues of the FVB/N-Trp53^em2Hwl^/Korl KO mice. Therefore, we believe that our results provide first evidence that the TALEN-mediated Trp53 mutant gene may successfully induce tumor formation in FVB/N mice; however, further studies are required to provide a detailed comparison. The survival rate and organ weight of FVB/N-Trp53^em2Hwl^/Korl and C57BL/6-Trp53^em1Hwl^/Korl KO mice differed from the results of previous studies. It has been reported that in p53 deficient mice with deletion of exon 2 and exons 2–6, the survival rates and thymus weight gradually decrease after age 10 weeks, regardless of their genetic background [11, 13]. Moreover, two types of p53 KO mice with deletion of intron 4 and exon 5 showed a decrease of survival rate at age 6–8 weeks and 15–25 weeks, as well as a lower thymus and testis weight compared with those of WT mice [9, 14]. Furthermore, in BALB/cByJ p53 deficient mice with exon 2–6 deletion, the survival rate was significantly decreased after 6 months of age, although lymphoma and sarcoma were detected in various tissues [12]. However, FVB/N-Trp53^em2Hwl^/Korl and C57BL/6-Trp53^em1Hwl^/Korl KO mice with exon 2 deletion of the Trp53 gene showed a rapid decrease in survival at age 8–9 weeks, while decrease of organ weight was only observed for the lung and spleen. We believe this disparity to be a result of differences in the deletion site of the target gene, as well as the pathological severity caused by animal genetic background and sensitivity.

Numerous studies have reported the correlation between the inactivation mechanism of p53 and tumorigenic phenotypes. Random mutations in the DNA binding domain of p53 stimulates the formation of tumors in several tissues including colon, breast, lung, brain and stomach by inhibiting the binding of p53 to specific DNA sequence [19, 20]. Deletion of carboxyl terminal domain of p53 prevents the formation of p53 tetramers, resulting in the formation of tumors in various tissues, while multiplication of the Mdm2 gene results in sarcoma and brain tumors by inducing the degradation of p53 [19, 21]. Also, lymphomas and tumors in the cervix and liver were induced by products of viral oncogenes in viral-infected mammalian cells, via inactivation and degradation of p53 [21]. The results of the current study determined that KO mice produced by TALEN-mediated deletion of exon 2 containing transactivation domain show defective Trp53 protein. Three types of tumors containing lymphoma, teratoma and sarcoma, were detected in several tissues. Therefore, we believe that the tumorigenic molecular mechanism of KO mice with the TALEN-mediated Trp53 mutant gene is associated with the condition of Trp53 inactivation and degradation, rather than the interference of DNA binding and the prevention of tetramer formation. However, more studies are required to clarify the mechanism of action.

In the current study, tumor formation on the skin surface was only observed in Trp53 deficient mice with the FVB/N background. Moreover, a similar tumor spectrum for both mice was detected with respect to malignant lymphoma, while teratoma and hemangiosarcoma were more developed in mice with an FVB/N background. In previous studies, the effects of genetic background on tumorigenesis have been analyzed in only a few Trp53-deficent mice. In the present study, tumor development was also monitored in Trp53 null mice of pure 129S1/SvImJ and C57BL/6 × 129S1/SvImJ mixed genetic background. The pure 129S1/SvImJ mice with the Trp53 null allele developed tumors faster than mice with mixed genetic backgrounds, although the effects were only observed during induction of aggressive teratocarcinomas [18]. Furthermore, Trp53-deficient mice (Trp53^+/−^ and Trp53^−/−^) and their wild-type littermates (Trp53^+/+^) from the two different genetic backgrounds (129S1/SvImJ and mixed C57BL/6 × 129S1/SvImJ) were monitored up to 2 years of age. The Trp53^+/−^ and Trp53^−/−^ 129S1/SvImJ mice show enhanced tumorigenesis rates compared to the C57BL/6 × 129S1/SvImJ mixed genetic background mice, although the tumor spectra were very similar in both mice [22]. The present results therefore give additional evidence that tumorigenesis under the conditions of Trp53 deficiency in TALEN-mediated Trp53 mutant genes correlate with genetic background. However, investigations using multiple strains of mice with the same deletion site of Trp53 gene are necessary to clarify the effects of genetic background during tumorigenesis.

Generally, genes of mammalian are passed from parents to offspring according to Mendel's law of heredity [23]. This principal has been applied to produce FVB/N-Trp53^em2Hwl^/Korl and C57BL/6-Trp53^em1Hwl^/Korl KO mice from heterozygous breeding pairs. But, the genotyping results of all mice from heterozygote mating was inconsistent with this law because the frequency of FVB/N-Trp53^em2Hwl^/Korl and C57BL/6-Trp53^em1Hwl^/Korl KO mice were lower at 19% and 14% than expected. In previous study, the homozygous mutant type of p53-deficient mice were founded as 23% frequency from 131 offspring although litter size were maintained with normal level [9]. Therefore, average 17% of KO frequency in our study were different from 23% of previous study. This difference was attributed to the difference in embryonic or neonatal lethality during pregnancy and delivery, even though further experiment will be needed to conform these level.

## Conclusions

The present study compared tumor development and phenotype between two variants of mice having TALEN-mediated deficiency of the Trp53 gene. Our results indicate that the deficiency of Trp53 using the TALEN-mediated Trp53 mutant gene accelerated tumorigenesis in FVB/N-Trp53^em2Hwl^/Korl and C57BL/6-Trp53^em1Hwl^/Korl KO mice, although their diversity was observed in the FVB/N-Trp53^em2Hwl^/Korl KO mice. However, further studies investigating the onset point and detection of prolonged clinical symptoms are required before these models can be applied to efficacy studies for potential cancer drugs.

## Methods

### Production and identification of FVB/N-Trp53^em2Hwl^/Korl and C57BL/6-Trp53^em1Hwl^/Korl KO mice

The 2 strains of Trp53 KO mice (FVB/N-Trp53^em2Hwl^/Korl and C57BL/6-Trp53^em1Hwl^/Korl) were produced by Professor Han-Woong Lee at the Mouse Molecular Genetics Lab, Department of Biochemistry, Yonsei University, Korea. To target the Trp53 gene in the mouse genome, the two most active TALENs specific to exon 2 of Trp53 were designed and synthesized (Fig. 1). After assessing their efficacy for small deletion at the target site in mouse NIH3T3 cells, each TALEN mRNA was injected into the cytoplasm of mouse pronuclear-stage embryos to produce mutant C57BL/6 and FVB/N founders (F0) with mutations in Trp53. The resultant Trp53 gene of FVB/N-Trp53^em2Hwl^/Korl KO mice has nucleotides 55 to 61 deleted (deletion of 7 bp), while that of C57BL/6-Trp53^em1Hwl^/Korl KO mice has the 56 nucleotide deleted (deletion of 1 bp).

The target gene was identified by DNA-PCR analysis of genomic DNA isolated from the tails of 3-week-old founder mice. The TALEN-mediated Trp53 mutant genes in FVB/N and C57BL/6 background strains were amplified using specific primer sets. The Trp53 WT gene in FVB/N and C57BL/6 mice was identified by the forward primer (5′-ATTTC CCTAC TGGAT GTCCC ACC-3′) and reverse primer (5′-TTTCC ATAAG CCTGA AAATG TCTCC TGG-3′). Furthermore, Trp53 mutant gene were identified by the forward primer (5′-TTCTC TCAGG CAAGG GGAGG ATA-3′) and reverse primer (5′-GAGCT CCCTC TGAGA CATT-3′) for FVB/N-Trp53^em2Hwl^/Korl KO mice as well as forward primer (5′-TCGAG CTCCC TCTGA GCA-3′) and reverse primer (5′-TTCTC TCAGG CAAGG GGAGG ATA-3′) for C57BL/6-Trp53^em1Hwl^/Korl KO mice. Thereafter, 10 pmol of sense and antisense primers were added, and the reaction mixture was subjected to 38 cycles of amplification on a T^100^ Thermal Cycler (Bio-Rad Com., Hercules, CA, USA) as follows: 30 s, 94 °C; 30 s, 62 °C; 45 s, 72 °C. After amplification, the final PCR products of 212 bp, 107 bp and 115 bp were electrophoresed on 2% agarose gels. The Trp53 WT gene was detected as 212 bp PCR products, while Trp53 mutant genes were identified as107 bp PCR products for FVB/N-Trp53^em2Hwl^/Korl KO mice and 115 bp PCR products for C57BL/6-Trp53^em1Hwl^/Korl KO mice (Additional file 1: Fig. S1).

### Animal care and use

The animal protocol used in this study was reviewed and approved in accordance with the protocols for ethical procedures and scientific care set by the Pusan National University-Institutional Animal Care and Use Committee (PNU-IACUC; Approval Number PNU-2016-1094). Adult FVB/N-Trp53^em2Hwl^/Korl and C57BL/6-Trp53^em1Hwl^/Korl KO mice were produced at the Mouse Molecular Genetics Lab, Department of Biochemistry, Yonsei University, Korea, and were kindly provided by Professor Han-Woong Lee. FVB/N and C57BL/6 mice were purchased from Samtako BioKorea Co. (Osan, Korea). All mice were handled at the Pusan National University-Laboratory Animal Resources Center accredited by the Korea Food and Drug Administration (KFDA) (Accredited Unit Number-000231) and The Association for Assessment and Accreditation of Laboratory Animal Care (AAALAC) International (Accredited Unit Number; 001525). Throughout the study, animals were provided with ad libitum access to water and a standard irradiated chow diet (Samtako BioKorea Inc.) consisting of moisture (12.5%), crude protein (25.43%), crude fat (6.06%), crude fiber (3.9%), crude ash (5.31%), calcium (1.14%) and phosphorus (0.99%). During the experiment, mice were maintained in a specific pathogen-free (SPF) state under a strict light cycle (lights on at 08:00 h and off at 20:00 h) and maintained at 23 ± 2°C and 50 ± 10% relative humidity.

The litter size of each pregnant female mouse was counted directly, and the total number and gender of pups at 3 weeks after birth was assessed and confirmed by measuring the anogenital distance at weaning after birth [24]. The genotype of both mice was not completely reflected as per the Mendelian inheritance, although their levels had large deviations. The WT, hetero type (HT) and KO ratio of FVB/N-Trp53^em2Hwl^/Korl KO mice was 1:1.31:0.53, while those of C57BL/6-Trp53^em1Hwl^/Korl KO mice was 1:1.87:0.47 (Additional file 1: Fig. S1). Furthermore, the litter size was higher in FVB/N-Trp53^em2Hwl^/Korl KO mice than C57BL/6-Trp53^em1Hwl^/Korl KO mice. The sex ratio of founder mice among FVB/N-Trp53^em2Hwl^/Korl and C57BL/6-Trp53^em1Hwl^/Korl KO mice was similarly maintained at 1.12 (male):1 (female) (Additional file 1: Fig. S1).

The phenotype analyses of two KO mice was based on the gold standard protocol of International Mouse Phenotyping Consortium (IMPC). In phenotyping protocols for IMPC pipeline, mice were firstly analyzed based on the various physiological parameters in life (4–16 weeks), and further analyses including clinical chemistry, histopathology, hematology and organ weight were carried out after the termination at 16 weeks.

### Measurement of body and organs weight

The body weight of FVB/N-Trp53^em2Hwl^/Korl and C57BL/6-Trp53^em1Hwl^/Korl KO mice was measured daily at 10:00 am from age 4 weeks to 16 weeks using an electrical balance. At 16 weeks of age, the weights of nine organs (brain, ovary (or testis), kidney, adrenal gland, spleen, liver, thymus, heart and lung) collected from the sacrificed mice were determined using the same method employed to detect the body weight.

### Observation of clinical signs, tumor growth and analysis of survival rate

Several pathological signs including dead animals, abnormal behaviors and formation of solid tumors were regularly recorded during all experimental study. In case of surface solid tumor, their maximum size were measured and recorded more than once a week from week 4 to 16. The human endpoint of KO mice was set when the tumor excessed 2 Cm in size in any direction. In case of internal tumor, their progression were basically measured by a change in body weight of mice with more than 10% within 1 weeks.

Also, the survival rate of mice in each group was then calculated using the following equation:

$${\text{Survival}}\;{\text{rate}} = {\text{KO}}\;{\text{mice}}\;\left( {{\text{or}}\;{\text{WT}}\;{\text{mice}}} \right)\;{\text{still}}\;{\text{alive}}\;{\text{for}}\;16\;{\text{weeks}}/{\text{total}}\;{\text{number}}\;{\text{of}}\;{\text{KO}}\;{\text{mice}}\;\left( {{\text{or}}\;{\text{WT}}\;{\text{mice}}} \right)$$

### Whole blood and serum analysis

At 16 weeks of age, all mice in each group were fasted for 8 h, after which blood was collected from the abdominal veins using a 1 mL syringe attached to a needle (21 SWG) following induction of anesthesia by intraperitoneal injection of Alfaxan (JUROX Pty Limited, Rutherford, Australia, 13 mg/kg body weight i.v.) [25, 26]. Also, cervical dislocation was performed to conform a death of mice following induction of anesthesia. Blood analysis and serum biochemistry were performed for all collected samples. Whole blood was placed in plain capped bottles containing ethylenediaminetetraacetate (EDTA), and the components were then analyzed using an automated cell counter (Beckman-Coulter Inc., USA) with standard calibration according to the manufacturer’s instructions. The levels of white blood cells (WBC), red blood cells (RBC), hemoglobin (HGB), hematocrit (HCT), mean corpuscular volume (MCV), mean corpuscular hemoglobin (MCH), mean corpuscular hemoglobin concentration (MCHC), corpuscular hemoglobin concentration mean (CHCM), corpuscular hemoglobin content (CH), hemoglobin concentration distribution width (HDW), platelets (PLT), and mean platelet volume (MPV) were measured in duplicate for each sample.

Serum was obtained for biochemical analysis by centrifuging the whole blood at 1500 × g for 10 min. Serum biochemical components, including alkaline phosphatase (ALP), alanine aminotransferase (ALT), aspartate aminotransferase (AST), total protein (TP), albumin (ALB), total bilirubin (Bil-T) blood urea nitrogen (BUN), creatinine (Crea), glucose (Glu), cholesterol (CHO), triglyceride (TG), calcium (Ca) and low density lipoprotein (LDH), were assayed using an automatic serum analyzer (Hitachi 747; Hitachi, Tokyo, Japan). All assays were measured in duplicate using fresh serum.

### Histological analysis

Tumor tissues were excised from FVB/N-Trp53^em2Hwl^/Korl and C57BL/6-Trp53^em1Hwl^/Korl KO mice, fixed in 10% formalin, embedded in paraffin wax, routinely processed, and then sectioned into 4 μm thick slices. The tumor sections were then stained with hematoxylin and eosin (H&E), after which they were examined by light microscopy (Leica Microsystems, Wetzlar, Germany) to detect alterations in histological structure. Tumor type was also identified by Pro. Beum Seok Han at the Department of Pharmaceutical Engineering, Hoseo University, Korea.

### Statistical analysis

Statistical analyses were performed with SPSS for Windows, release 10.10, standard version (SPSS, Inc., Chicago, IL, USA). One‑way analysis of variance followed by Tukey's post hoc test for multiple comparisons was performed to identify significant differences between groups. Data were presented as mean ± standard deviation. A *P* value of < 0.05 was considered significant.

## Supplementary Information

**Additional file 1: Fig. S1.** Genotype and sex ratio of (**A**) FVB/N-Trp53^em2Hwl^/Korl and (**B**) C57BL/6-Trp53^em1Hwl^/Korl KO mice. The genotype of wild type (WT), hetero type (HT) and knockout homo type (KO) were identified by DNA-PCR analysis of genomic DNA. Overall, 30 mice produced from mating between HT male and HT female mice were analyzed. Values are expressed as the means ± SD.

## Data Availability

The datasets used and/or analyzed during the current study are available from the corresponding author on reasonable request.

## Abbreviations

ALT: Alanine aminotransferase
ALB: Albumin
ALP: Alkaline phosphatase
AST: Aspartate aminotransferase
AAALAC: Association for Assessment and Accreditation of Laboratory Animal Care
SPF: Specific pathogen-free
Bil-T: Total bilirubin
BUN: Blood urea nitrogen
Ca: Calcium
CHO: Cholesterol
Crea: Creatinine
CH: Corpuscular hemoglobin content
CHCM: Corpuscular hemoglobin concentration mean
EDTA: Ethylenediaminetetraacetate
GEM: Genetically engineered mice
Glu: Glucose
HT: Hetero type
HGB: Hemoglobin
HCT: Hematocrit
H&E: Hematoxylin and eosin
HDW: Hemoglobin concentration distribution width
IMPC: International Mouse Phenotyping Consortium
EDTA: Ethylenediaminetetraacetate
KO: Knockout
KFDA: Korea Food and Drug Administration
LDH: Low density lipoprotein
MCH: Mean corpuscular hemoglobin
MCHC: Mean corpuscular hemoglobin concentration
MCV: Mean corpuscular volume
MPV: Mean platelet volume
PLT: Platelets
RBC: Red blood cells
SPF: Specific pathogen-free
TALEN: Transcription activator-like effector nucleases
TG: Triglyceride
TP: Total protein
WBC: White blood cells
