# Supplementary figures and images for: Sensitivity to tumor development by TALEN-mediated Trp53 mutant genes in the susceptible FVB/N mice and the resistance C57BL/6 mice
Source: Lab Anim Res. 2021 Nov 29;37:32. doi: 10.1186/s42826-021-00107-y (PMC8628475; doi:10.1186/s42826-021-00107-y)

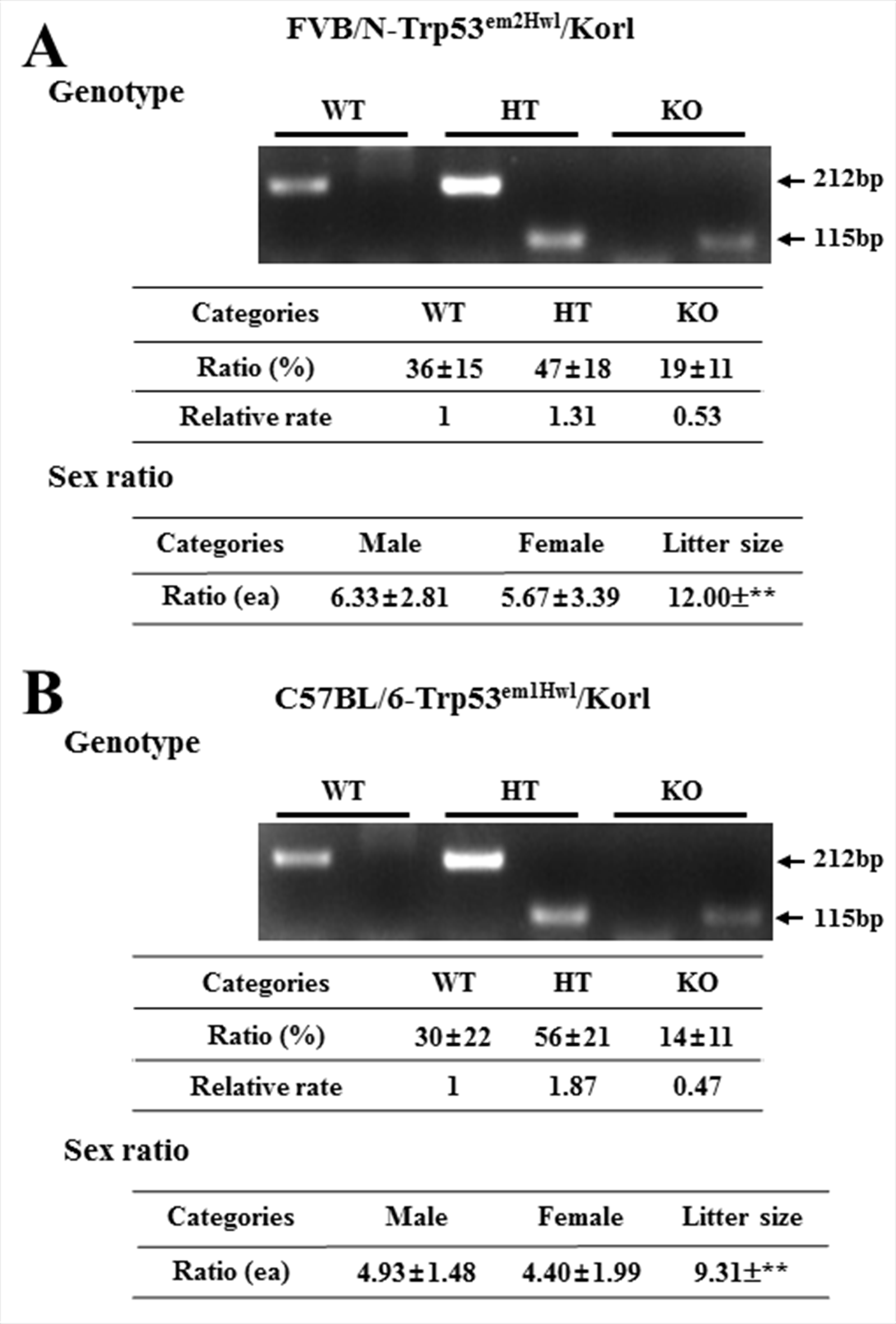

Supplement: Supplementary file 1 — Additional file 1: Fig. S1. Genotype and sex ratio of (A) FVB/N-Trp53em2Hwl/Korl and (B) C57BL/6-Trp53em1Hwl/Korl KO mice. The genotype of wild type (WT), hetero type (HT) and knockout homo type (KO) were identified by DNA-PCR analysis of genomic DNA. Overall, 30 mice produced from mating between HT male and HT female mice were analyzed. Values are expressed as the means ± SD. [file 42826_2021_107_MOESM1_ESM.tif]
